# Emergency care for avalanche buried patients - a narrative review

**DOI:** 10.1186/s13049-026-01543-2

**Published:** 2026-02-20

**Authors:** Giacomo Strapazzon, Oyvind Thomassen, Christopher Van Tilburg, Kyle McLaughlin, Sven Christjar Skaiaa, Hermann Brugger, Mathieu Pasquier

**Affiliations:** 1 Mountain Clinic, Eurac Research, Via Ipazia 2, Bolzano, 39100 Italy; 2 Department of Medicine - DIMEM, University of Padova, Padova, Italy; 3 Corpo Nazionale del Soccorso Alpino e Speleologico - CNSAS, Milano, Italy; 4 Medical Commission, International Commission for Alpine Rescue - ICAR MedCom, Zurich, Switzerland; 5 Department of Anaesthesia and Intensive Care, Haukeland University Hospital, Bergen, Norway; 6 Mountain Medicine Research Group, Norwegian Air Ambulance Foundation, Bergen, Norway; 7 Department of Emergency Medicine, Occupational and Travel Medicine, and Mountain Clinic, Providence Hood River Memorial Hospital, Hood River, OR USA; 8 Mountain Rescue Association, San Diego, CA USA; 9 Department of Emergency Medicine, Canmore General Hospital, Canmore, Canada; 10 University of Calgary Cumming School of Medicine, Calgary, Canada; 11 Division of Prehospital Services, Air Ambulance Department, Oslo University Hospital, Oslo, Norway; 12 Department of Emergency Medicine, Lausanne University Hospital and University of Lausanne, Lausanne, Switzerland

**Keywords:** Asphyxia, Avalanche, Extracorporeal Life Support, ECLS, Extracorporeal Membrane Oxygenation, ECMO, Emergency Medical Services, EMS, Helicopter Emergency Medical Services, HEMS, Hypercapnia, Hypothermia, Hypoxia, Search and Rescue, SAR, Snow

## Abstract

Avalanches claim the lives of around 160 winter recreationists and workers in hazardous snow-covered mountain regions worldwide. These fatalities result from asphyxia, trauma, and hypothermia. Survival for critically buried subjects relies on the speed of extrication, absence of trauma, presence of a patent airway and air pocket, immediate field treatment, and rapid transport to the most appropriate hospital by organized search and rescue (SAR) and emergency medical services (EMS). Survival may be improved with helicopter EMS, advancements in safety and rescue equipment technology, early deployment of professional SAR teams with checklists and standard operating procedures (SOPs), and the use of drones. Prehospital and in-hospital care of an avalanche accident requires medical, technical, logistical, and organizational competencies.

This narrative review of historic and updated survival curves, professional society guidelines, and snow burial studies, discusses the best practices for prehospital care, triage, transport, and in-hospital management of avalanche patients.

**Supplementary Information:**

The online version contains supplementary material available at 10.1186/s13049-026-01543-2.

## Introduction

Avalanches claim the lives of around 160 winter recreationists and workers who operate in hazardous terrain in snow-covered mountain regions of Europe and North America [1]. More fatalities likely exist with unreported accidents in other continents [2]. Asphyxia, related to hypoxia and hypercapnia, is the main cause of death (65–100%), followed by trauma (5–29%) and accidental hypothermia (0–4%) [3, 4]. Survival depends mainly on the timely extrication of critically buried avalanche subjects, usually by companions or bystanders before organized search and rescue (SAR) and emergency medical services (EMS) arrive. On-scene management of an avalanche accident requires medical, technical, logistical, and organizational competencies [2, 4, 5]. SAR and EMS providers need to gather information on the duration of critical burial, core temperature, vital signs, airway status, and the presence of an air pocket at extrication. The implementation of evidence-based recommendations and the use of an avalanche resuscitation checklist [2, 4, 6] have improved the quality of on-scene management and the choice of destination hospital [4, 6, 7]. Regional and local protocols should be tailored to each SAR and EMS system. These protocols should include transport to hospitals capable of extracorporeal life support (ECLS) or trauma care, when indicated, and the implementation of standard operating procedures (SOPs) [8, 9]. In-hospital triage and indication for ECLS rewarming in cardiac arrest (CA) patients should rely on prognostic markers and triage tools [10, 11].

**Fig. 1:**
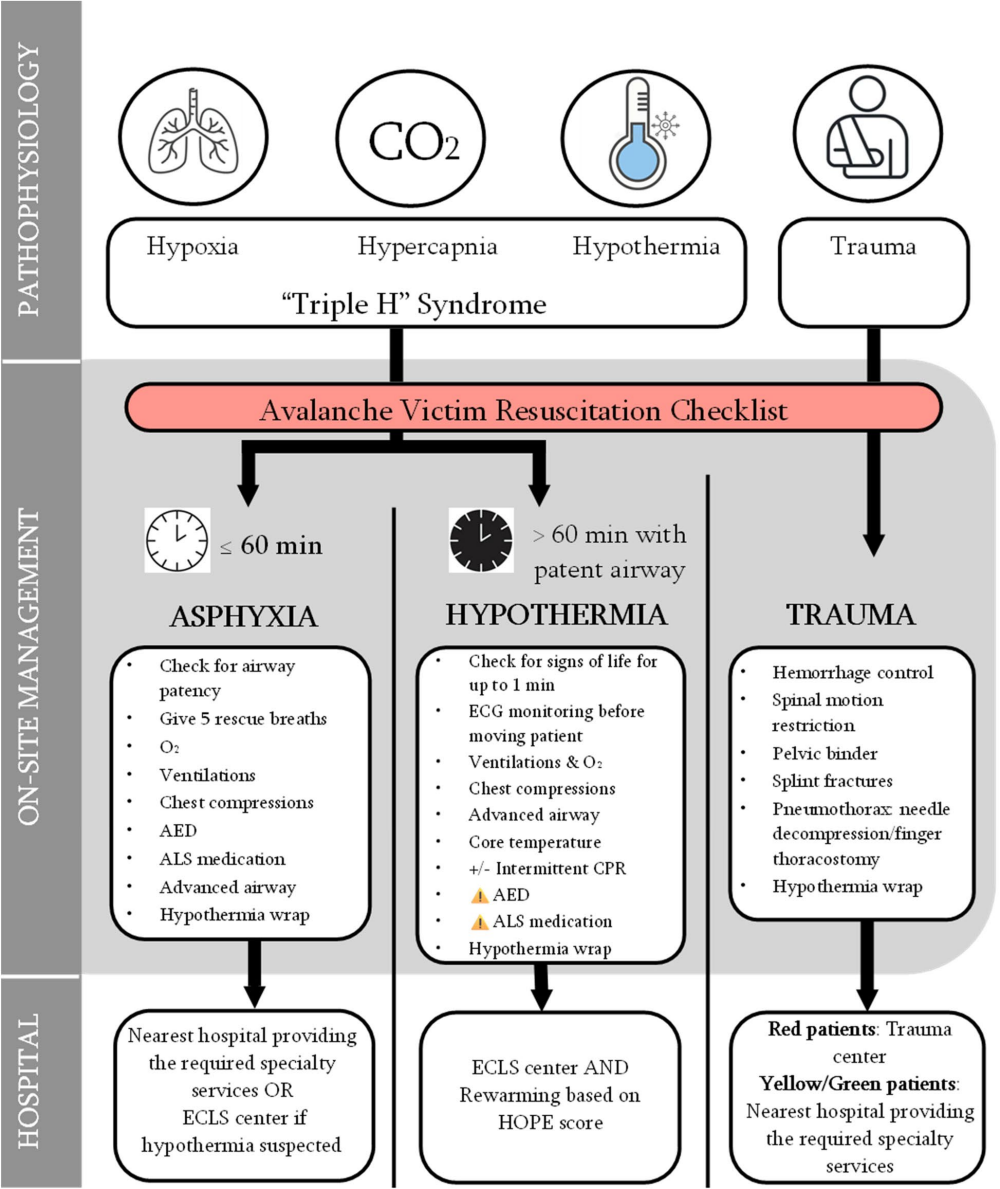
Avalanche management visual summary. ALS: Advanced Cardiovascular Life Support; AED: Automated External Defibrillator; CA: cardiac arrest; CPR: cardiopulmonary resuscitation; ECG: electrocardiographic monitoring; ECLS: extracorporeal life support; HOPE: Hypothermia Outcome Prediction score after ECLS. In the triage method used in this figure, “Red patients” refer to patients with a life-threatening emergency who needs immediate care. “Yellow/Green patients” refer to patients with not immediately life-threatening or minor injuries who can wait longer for care

This narrative review focuses on the emergency care for avalanche patients, discussing current evidence and potential pitfalls during prehospital and in-hospital care. By addressing current organizational realities and proposing future models to standardize procedures, improve training, and enhance knowledge application, we aim to improve the management of avalanche patients on-scene and in hospitals (Fig. 1).

## Evidence from survival curves and pathophysiological studies

**Fig. 2:**
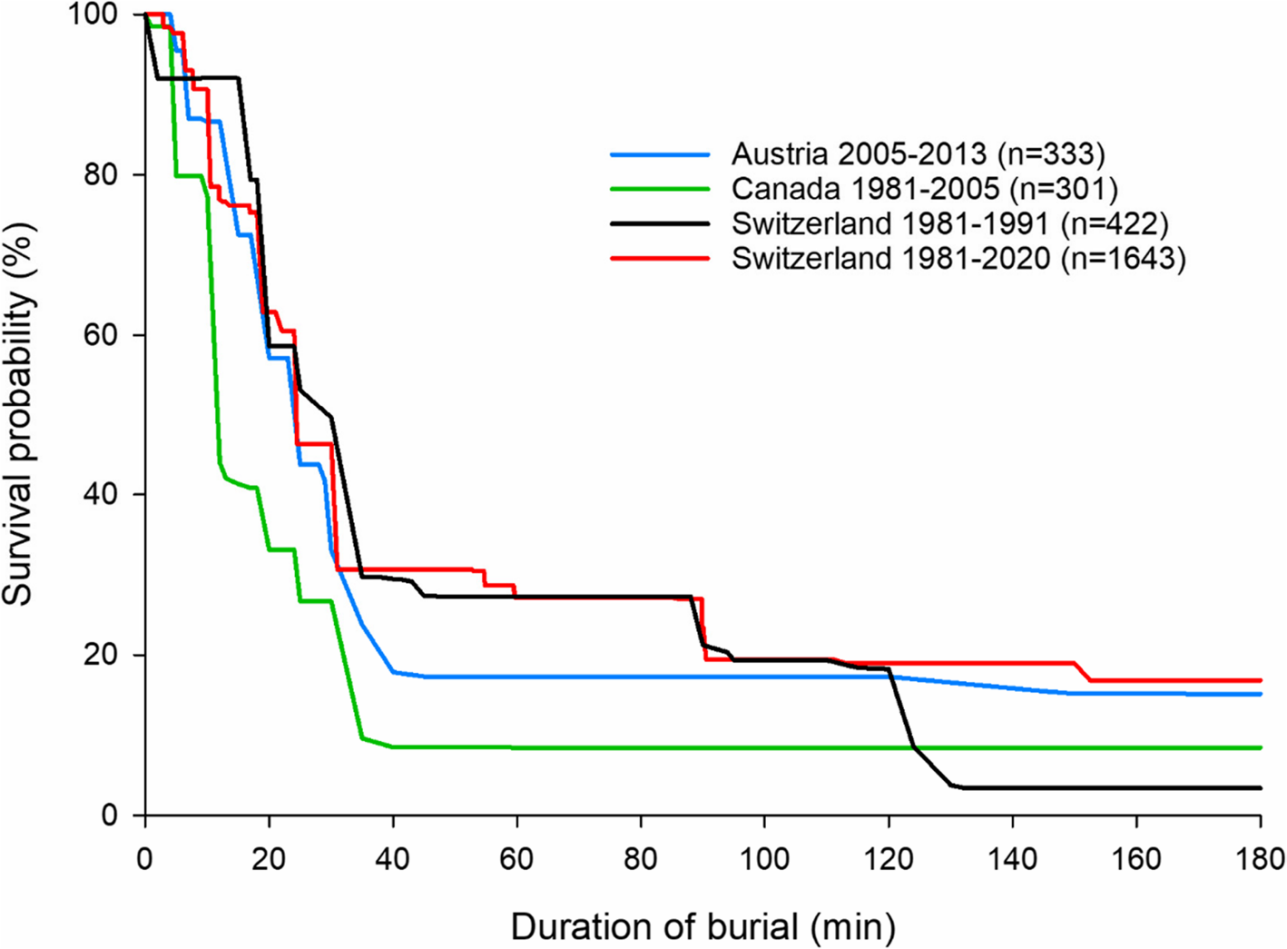
Survival curve for critically buried avalanche subjects. Survival curves for critically buried subjects from Austria (blueline; 333 subjects between 2005 and 2013), Canada (green line; 301 subjects between 1981 and 2005) and Switzerland (black line; 422 subjects between 1981 and 1991; red line; 1643 subjects between 1981 and 2020). Modified from Falk et al., 1994; Haegeli et al., 2011; Procter et al., 2016; and Rauch et al. 2024 with permission [13, 14, 16, 19].

The grade of burial (“critical” if the head and chest are buried vs. “partial” if the head and chest remain free) is the main determinant of survival. Most subjects without critical burial were alive at extrication: 96% compared with 48% of those with critical burial [12]. Survival of critically buried subjects is time dependent. The first survival analysis based on the Turnbull algorithm was published in 1994 from Swiss avalanche accidents [13]. The survival function showed a survival probability as high as 93% until 15 min after burial (“survival phase”), followed by a steep drop to about 30% at 35 min (“asphyxia phase”), a plateau in survival until 90 min (“latent phase”) and a second drop to 3% at about 90 min (Fig. 2). Compared to the first survival analysis, the updated survival rate based on four decades increased from 43% to 53% [14]. This improvement can be attributed to the decrease in median rescue time from 45 min to 25 min and the increase in survival rates among subjects rescued by SAR and EMS providers from 14% to 23%. However, survival probability begins to decline sharply after just ten min of burial. Survival curves from avalanche accidents from different countries showed similar mortality rates, but slightly different time courses (Fig. 2) [12, 13, 15, 16]. In Canada, there was an earlier drop in survival due to more trauma deaths and higher snow densities in areas with a maritime climate [16]. A greater burial depth is associated with lower survival, independently from the burial duration [15]. Burials in higher density snow packs are at increased risk of developing normothermic CA by asphyxia because of insufficient oxygen supply and excessive carbon dioxide accumulation [17]. Although no data are available yet, survival curves may change over the coming decades as snow properties may be altered with climate change [18].

A patent airway is essential for survival of critically buried avalanche subjects [20]. The time to normothermic CA is variable, but no subjects who underwent CA after a short (≤60 min) burial survived with good neurological outcomes when burial duration was >20 min [4, 20, 21]. Even if the airway is patent, short burial does not guarantee survival. Asphyxia can result also from inadequate gas diffusion in the surrounding snow, or from compression of the chest by avalanche debris that hinders ventilation and alters the cardiovascular function [5].

Experimental studies have shown that the presence of an air pocket delays the onset of hypoxemia in proportion to its size [15, 17, 22–25]. Similarly, respiratory gases diffuse more readily in lower-density snow, which also delays the onset of hypoxemia [17, 23, 24]. Buried subjects experience rapid accumulation of carbon dioxide within few minutes with a strong ventilatory response and a significant dyspnea [3]. Using an air diverter (also called an artificial air pocket device) that moves exhaled CO_2_ away from the face or a device that enhances airflow to the subjects’ airways [26–28] could increase survival rates by extending the time window to hypoxemia, hypercapnia and successful rescue.

After an avalanche burial of 1 h, subjects can reach a core temperature <30°C [4] but the cooling rate is variable [28–31]. Cooling rates up to around 10°C/h have been reported in patients who survived [4]. Despite being typically younger and healthier than urban hypothermic patients, the survival rate for avalanche patients with out-of-hospital hypothermic CA that undergo extracorporeal rewarming is only 12% [32–35]. Experimental and epidemiological studies have shown that asphyxia and not hypothermia is the responsible factor for the considerably lower survival rate among avalanche patients [3, 19, 23].

## Prior to the avalanche rescue mission

SAR teams, healthcare providers, and dispatchers should be familiar with avalanche patient management, triage algorithms and safety procedures [2, 4, 36]. SAR and EMS organizations should be transparent about their providers’ capabilities and operational limitations (e.g., access to exposed alpine terrain) to enable the dispatch center to deploy an appropriate response.

All providers should be self-sufficient in winter mountain conditions and be competent to perform national and regional clearly defined SOPs. The required personal protective equipment and emergency gear should be defined within each organization and rescuers should be trained in its use. In addition to the standard shovel, probe and transceiver [37, 38], rescuers are recommended to use avalanche airbag backpacks. These backpacks reduce the risk of critical burial by 27% when inflated [39]. They may also consider the combination with an air diverter or air supplier to increase occupational safety margins [2, 26–28], although this technology is new and may not be available. Medical equipment and medications should be protected from cold and electronic equipment should have full batteries [40].

When notified of an avalanche accident, the receiving dispatch center should follow a protocol to collect essential information without delaying companion rescue efforts. This includes, but is not limited to, the accident location, the weather and visibility conditions, the number of subjects (visible and/or buried), whether the buried subjects are equipped with transceivers, the number of rescue resources on-scene and any apparent risks to the callers or SAR personnel (Table 1). Once sufficient information has been gathered to initiate an appropriate first response, the caller should resume searching and provide first aid without delay, as subjects extricated by companion rescue have a greater survival compared to organized SAR [41]. If available, HEMS helicopters should be dispatched to shuttle SAR teams to the avalanche scene.

**Table 1:** Initiation of organized avalanche rescue by the dispatch center and emergency medical services

**Essential information at alarm:**
Location
Wheather conditions
Time of the avalanche
Number of injured/buried subjects
Buried subjects have transceivers (yes/no)
Immediate dangers or risks to bystanders and/or rescuers
If few resources, the caller is encouraged to continue searching or providing lifesaving first aid without further delay. An appropriate response is dispatched immediately.
**Supplemental information if enough resources or when contact is re-established:**
• Status on buried subjects and bystanders • Weather and visibility • Terrain and obstacles (air) • Activity type prior to the avalanche • Access routes for ground rescue • Identity of person still buried to retrieve International Mobile Equipment Identity (IMEI) and International Mobile Subscriber Identity (IMSI) on the subject’s mobile phone *

Additional resources are dispatched or withdrawn as needed

^*^Only applicable in countries utilizing mobile phone localization systems

**Fig. 3:**
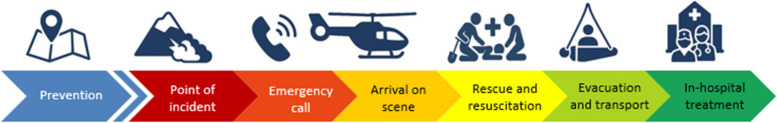
Avalanche chain of survival. When prevention has failed, the complexity of the phases in organized avalanche rescue depends on the number of buried subjects, weather, terrain, accessibility for providers and equipment, and possibilities for evacuation and transport. This dynamic situation must be continuously reevaluated. Resources are alerted (or retracted) as needed to ensure a balanced response and an intact chain with forward momentum

Studies show that most avalanche-buried subjects are located and extricated before organized rescue teams arrive. Only 5.5% of cases in Austria and 21% in Norway required localization by the organized SAR teams [41, 42]. Because injuries occur in about half of cases and mass casualty incidents (MCIs) are common, sufficient numbers SAR resources, including HEMS, should be dispatched to the scene [4, 41, 42]. Both the dispatch center and deployed providers must continuously evaluate whether the avalanche chain of survival remains intact (Fig. 3**).**

Operational and cognitive preparation for medical management of avalanche patients should initially prioritize the scene safety, efficient search and excavation strategies and the treatment of more common injuries (i.e., normothermic CA, trauma, and accidental hypothermia) (Table 2). The establishment of leadership should be part of the SOP, as well as a dynamic safety plan that should be continuously revised based on a risk assessment of further avalanche activity [2].

**Table 2:** Practical aspects of the prehospital management of a critically buried avalanche subject

**PRACTICAL ASPECTS FOR HEALTCARE PROVIDERS**	**RATIONALE**	**REFERENCE**
**Generalities**		
Try to be ready (incl. material) to manage the subject as soon as the face is exposed.	The initial phase of an avalanche accident is usually chaotic and dedicated to the localization and extrication of subjects. The medical management involves competencies and equipment that should be available on site.	[43]
Use a checklist.	Management of patients in cardiac arrest (CA) has specific recommendations. Use of a checklist is recommended and may improve management.	[2, 4, 43, 44]
Suspect severe trauma in avalanches in steep terrain with rocks and trees, and follow international trauma guidelines.	Chest and head trauma are the most frequent injuries in avalanches. Spinal, abdominal, and limb injuries are less frequent.	[3, 4, 45]
Reduce further heat loss using hypothermia wrap.	When the patient is extricated from the snow, it is at risk of higher heat loss due to convection.	[35]
**Short (≤60 min) burial**		
Be ready to bag-mask ventilation as soon as the face is exposed.	For subjects with burial time of ≤60 min without signs of life, presume asphyxia and provide rescue breaths as soon as possible.	[4]
Supraglottic airway device may be an option during extrication.	Face is exposed usually first, but more shovelling is needed to free and extract the subject totally from the snow.	[2, 4]
Chest compressions can be provided effectively even in atypical position before complete extrication.	Completely extricating a subject for resuscitation in standard position from 1-m burial depth can takes five to twenty min.	[46]
**Long (>60 min) burial**		
Be ready to assess for airway patency and the presence of an air pocket as soon as the face is about to be exposed.	If the burial time is >60 min, airway patency and the presence of an air pocket should be determined when the face is exposed.	[4]
Be ready to clinically evaluate the patient as soon as the face is exposed.	For subjects buried >60 min, carefully check for signs of life, including vital signs, for up to one minute.	[4]
Anticipate having a defibrillator on site for the moment the subject will be accessed and place defibrillation pads before moving the subject.	For subjects buried >60 min without signs of life, electrocardiographic (ECG) monitoring, ideally using defibrillator pads ready to defibrillate, should be started as soon as the thorax is accessible and ideally before moving the patient.	[4, 47, 48]
Anticipate having an esophageal thermometer (if available) on site for the moment the subject will be accessed, in order to measure core temperature in CA patients. The target position of the tip of the probe is the lower third of the esophagus.	Esophageal temperature with the tip of the probe inserted into the lower third of the esophagus is the preferred method of core temperature measurement in subjects in CA or with a secured airway.	[49, 50]
The target position of the tip of the probe is the lower third of the esophagus. In an adult patient of average size, this corresponds to a distance of approximately 40 cm from the incisors.	Placing the probe too proximally, such as behind the trachea, may yield inaccurate readings because inhaled cold air, resulting in falsely low values.	[49, 50]
Resuscitation should not be attempted on subject with an obstructed airway in asystole, who have been buried for >60 min.	Only one avalanche patients survived with an unwitnessed asystolic CA following a long critical-burial.	[4, 7, 20, 51]

^*^Whatever the burial duration, the rescuer with the highest medical/paramedical background should be present when the face is exposed

## Prehospital patient assessment and management

Avalanche rescue SOPs begins with establishing leadership and securing the scene. Companions, or professional rescuers, should initiate a surface search, transceiver search, pinpoint search, and shovelling to locate and extricate buried subjects.

This initial phase of medical prehospital care requires considerable anticipation and the immediate availability of suitable equipment. SAR team personnel should, therefore, rotate shovelling and extrication duties to prevent exhaustion [2] and ideally preserve ALS providers for direct patient care. Initial medical care on-scene generally follows X-ABCDE (X, external bleeding; A, airway; B, breathing; C, circulation; D, disability; E, environment and evacuation) [7, 52, 53]. Medical care, specifically resuscitation, should commence as soon as practically feasible once the face and chest have been exposed.

### Airway assessment

It is essential to check for airway patency and the presence of an air pocket when the face is exposed, especially if the burial time is >60 min [4]. The terms obstructed and blocked airway require that both the mouth and nose be completely filled with compacted snow, debris or vomitus. An air pocket is any snow-free space in front of the mouth and nose in a patient with a patent airway [4, 15, 54].

### Trauma

Severe trauma should be suspected in avalanches in steep terrain, especially with trees and rocks. Chest and head trauma are the most frequent injuries in avalanches [55, 56]. Resuscitation should be withheld in the presence of obvious signs incompatible with life, such as complete freezing, fixed chest rigidity, or lethal traumatic injuries [57]. Several studies show that the likelihood of a positive outcome following traumatic CA is very low [58, 59]. When severe trauma is suspected, on-scene trauma treatment should be started as soon as possible according to international trauma guidelines, considering spinal motion restriction due to environmental and logistical factors [45, 60, 61].

### Cardiac arrest

If the subject is in cardiac arrest, one should consider initiating CPR. For burials ≤60 min, CA is likely due to asphyxia rather than hypothermia. Resuscitation with airway management and ventilation should commence as soon as possible if burial duration is ≤60 min [4]. When evaluating the airway, rescuers should clear a blocked airway and, if the patient is apneic, commence rescue breathing. If the patient is pulseless, they should start chest compressions and attach an automatic external defibrillator, if available. Chest compressions can be provided effectively even in atypical positions prior to complete unburial, reducing the time to resuscitation [46]. Consider termination of resuscitation of CA patients with short burial or core temperature ≥30°C, according to the local protocols [2, 4, 7].

### Hypothermia

Hypothermic cardiac arrest may occur in burials >60 min and resuscitation is indicated if there is a patent airway. [2, 4]. An automatic external defibrillator should be utilized if one is available [62]. Electrocardiographic (ECG) monitoring, if available, should be started as soon as the chest is accessible and ideally before moving the subject [4]. Rescue collapse is witnessed CA in the setting of hypothermia. If hypothermic CA occurs after extrication in the context of a long burial duration, the prognosis may be more favourable than CA from trauma and asphyxia [33].

For cardiac arrest, if advanced life support (ALS) personnel are on-scene, early consideration should be given to endotracheal intubation, which is preferable over a supraglottic airway due to decreased likelihood of being dislodged during transport and improved airway protection [2, 4]. Providers should consider the limitations of airway equipment in the cold and visualization of the larynx in bright light during advanced airway management [63]. Obtaining a core temperature is recommended as it is useful for medical decision making but may be difficult to obtain in the field [2, 4]. Core temperature is most accurately measured using an esophageal probe, but epitympanic temperatures can be employed (with a thermistor probe and insulating headset cover) in subjects who are not fully conscious and without airway protection [4, 49, 64].

The use of the Avalanche Victim Resuscitation Checklist (Supplementary Fig. 1S) improved compliance of patient management from 59% to 95% and led to complete documentation of the required information in a Swiss HEMS [44].

Vital signs and core temperatures are positively correlated, and the level of consciousness can be used to estimate the degree of hypothermia as proposed in the Revised Swiss System [65]. The Revised Swiss System is reliable only if the patient does not have a coexisting condition affecting the level of consciousness, such as intoxication or traumatic brain injury.

### Patient packaging

After being extricated, the subject should be protected from cold using insulating material to reduce conductive losses and a vapor barrier to reduce convective losses. [35, 66]. Prolonged-burial avalanche patients with a pulse should be moved gently, kept horizontal, and have cardiac monitoring as disturbances, especially movement of the extremities, might precipitate rescue collapse from ventricular fibrillation or asystole [35, 47, 66].

### In field triage

Evacuation triage in avalanche prioritizes patients based on the presence of vital signs and burial time (Fig. 1). For subjects with short burial (≤60 min), or those with long (>60 min) burial but who are responsive, have a core temperature ≥30°C, and are hemodynamically stable with a systolic blood pressure >90 mmHg, no bradycardia (<45 bpm) and no ventricular arrhythmias, admission to the nearest hospital providing the required specialty services is reasonable [4, 7]. Trauma patients with a core temperature ≥30°C should be transferred to the nearest trauma center while patients with a core temperature <30°C should be transported to an ECLS center. Trauma should be considered as a likely cause of witnessed CA for subjects buried ≤60 min or with a core temperature ≥30°C. Asphyxia should be considered as a likely cause for unwitnessed CA, especially in the case of short burial. Rescue collapse from hypothermia should be considered as a likely cause of CA in subjects buried >60 min and with patent airway or patients who have witnessed cardiac arrest after extrication. The Avalanche Victim Resuscitation Checklist assists SAR and healthcare teams adhere to recommended medical protocols on-scene and facilitate transport decision from the avalanche site to the appropriate hospital (Supplementary Fig. 1S) [4, 6, 7].

### Transport

Air transport is the transport method of choice for subjects that are critically injured or in CA (Fig. 1). The preferred hospital, depending on the determined level of care, can usually be reached directly. Life-saving procedures are often performed before flight, notably in case of hoist and longline operations [67]. Mechanical chest compression devices and automated ventilators can be utilized in-flight to allow for improved diagnostic (i.e. ultrasound) or therapeutic (warm fluids or transfusions) interventions, or monitoring (i.e. blood sugar levels, temperature). If possible, active rewarming (e.g. electric heating blankets) should continue throughout the flight.

If evacuation by air is impossible (e.g. in remote areas, or in challenging weather conditions), ground evacuation may be the only viable option [68, 69].

SAR teams might face the dilemma of whether to remain on-scene awaiting ALS teams or to initiate immediate transport [68, 69]. Throughout the transport, critical life-saving interventions, such as hemorrhage control, airway/ventilatory assistance, and resuscitation must be maintained without interruption. Clear communication between on-scene rescuers and ALS providers is essential to decide whether continued on-scene management or rapid evacuation will provide the greatest benefit based on the patient’s injuries, management needs, and physiological status.

Basic life support (BLS) interventions, specifically manual cardiopulmonary resuscitation (CPR), have been demonstrated to be feasible during field transport in both studies and case reports [70–72]. For patients in suspected hypothermic CA with a core temperature <28°C, the principles of intermittent CPR (iCPR) may facilitate transport when continuous chest compressions are not operationally possible [7, 72].

Prior to initiating transport, the team should be organized to ensure adequate patient monitoring (visual, verbal, and, if available, medical monitoring devices) with regular reassessments and quality control of ongoing interventions. Care should be taken during packaging of the patient to avoid hypothermia, rescue collapse and secondary traumatic injury during transport, especially over uneven terrain.

For ALS providers, hands-free solutions such as automated ventilators and mechanical chest compression devices can improve transport efficiency and safety while maintaining high-quality treatment [73–75]. Automated ventilators also help reduce oxygen consumption, which may be crucial during prolonged transports with limited supply. Advanced diagnostic procedures (e.g. ultrasound) should only be performed when they directly inform decision-making or improve monitoring [45].

Logistical planning is crucial in case of ground evacuation to balance speed and safety, considering evacuation routes, avalanche risk, and weather. Prolonged, physically demanding evacuations in harsh conditions may pose significant cognitive and physical strain on rescuers, underlining the need for team rotation and fatigue management during extended operations [63, 76, 77]. Coordination with the dispatch center and receiving facilities can optimize outcomes and minimize delays. Clear role distribution within the SAR team and healthcare providers ensures uninterrupted delivery of critical interventions, structured documentation, and effective communication during handover.

## In-hospital care

Hospital management of avalanche subjects includes resuscitation and ECLS, rewarming, comprehensive trauma evaluation, intensive care, surgical interventions, psychiatric follow-up, and rehabilitation. Such care may require multidisciplinary collaboration across several specialties, coordinated by clinicians familiar with both trauma and hypothermia management (Table 3). The injury spectrum following avalanche accidents differs from typical trauma scenarios. The combination of asphyxia, multi-trauma, and hypothermia might be more complex than isolated injuries or primary CA (Fig. 1).

**Table 3:** Practical aspects of the in-hospital management of a critically buried avalanche subject

**PRACTICAL ASPECTS FOR HEALTCARE PROVIDERS**	**RATIONALE**	**REFERENCE**
**Before patient arrival**		
Proactive approach to gathering information, especially on the patient’s status of consciousness, duration of burial, vital signs, temperature, airway status and air pocket.	Time to gather authentic information from the accident site will probably be missing at the patient’s arrival at hospital. Work overload can result in missing or insufficient information from the accident scene.	[43, 54]
Assess whether extracorporeal life support (ECLS) should be considered.	Assembling the ECLS team may take some time.	[78]
Anticipate the need for blood products.	There may be a delay in the preparation of blood products.	[79]
Set up and activate material for external rewarming.	A large proportion of avalanche patients are cold-stressed or hypothermic, and temperature often continues to decrease during transport.	[3]
**Following patient arrival**		
Drawn blood for a potassium test from a central vessel (ideally a central venous line) to get an accurate sample, avoiding falsely high measurements due to haemolysis. Be aware of the usage of depolarizing neuromuscular paralytics (e.g. succinylcholine) that may increase the serum potassium level.	Serum potassium concentration predicts brain hypoxia on computed tomography after avalanche-induced cardiac arrest	[4, 80]
Predict successful rewarming including the estimation of the survival probability using the Hypothermia Outcome Prediction after ECLS (HOPE) score.	The HOPE score outperforms serum potassium as a triage tool to determine whether to rewarm hypothermic patients in CA, using a cut off of 10%.	[4, 7, 10, 81]
Perform a structured trauma and medical examination.	The injury spectrum following avalanche accidents differs from typical trauma scenarios. The combination of asphyxia, multi-trauma, and hypothermia is more complex than isolated injuries.	[4]
Delay trauma computed tomography in hemodynamically stable patients until partial rewarming has been achieved.	Several cases of circulatory collapse have occurred in the computed tomography scanner.	Expert consensus.
Assess early accurate core temperature measurement (esophageal, thermistor-base epitympanic, bladder, rectal).	Tympanic-based thermometers are most often used in prehospital, non-intubated patients; however, accurate temperature measurement is essential for guiding treatment and prioritization.	[49, 82]
Consider performing a chest X-ray or lung ultrasound in critically buried patients.	Negative pressure pulmonary edema has been described in critically buried avalanche subjects, even after short burial.	[83, 84]

### In-hospital management of avalanche patients not in cardiac arrest

Hospitals near avalanche terrain must have contingency plans to support the dispatch center in directing patients to the appropriate facility without delay, while also addressing potential needs for stabilization before transfer for definitive treatment. Many avalanche patients present as normothermic trauma patients who do not require a hospital with ECLS. Avalanche-buried patients with spontaneous circulation should be managed as standard trauma patients, with the critical addition of early core temperature measurement. Core temperature, which is often not assessed prehospitally, should be measured [2, 4]. Importantly, patients are frequently colder than initially reported due to afterdrop and further cooling during transport. In awake patients, thermistor-based tympanic probes - which are however not frequently available in hospitals - are recommended, while esophageal probes are preferred in intubated patients [4, 45, 49, 50, 85]. Bladder temperature measurement also allows for continuous temperature measurement [35, 85]. If core temperature is <30°C, the team must be alerted to the heightened risk of arrhythmias [4, 7]. If required, central venous access should not be inserted by jugular or subclavian route, but rather by femoral route, to avoid arrhythmias and other complications. Point of care ultrasonography can be used at the bedside however trauma computed tomography may be delayed in hemodynamically stable patients until partial rewarming has been achieved. Non-urgent surgeries may be postponed in deep hypothermia cases until normothermia is achieved, due to the increased arrhythmia and bleeding risk. If induction of anesthesia is needed, heat loss should be prevented using active rewarming methods, as core temperature typically decreases because of internal heat redistribution [86]. Hypothermia-induced bradycardia and hypotension are the consequences of the reduced metabolic rate and do not require specific treatment [8, 35].

Patients may continue to cool even in the in-hospital setting including during short transports or seemingly minor procedures [87]. Passive and active rewarming is indicated for all hypothermic patients, including those with cold stress [4, 7, 35]. Available methods include chemical or electric heating blankets, forced-air warming, warmed intravenous fluids [8, 35, 66], intravascular catheter and renal replacement therapy (e.g. hemodialysis and hemofiltration) [88, 89]. Chemical and electric blankets alone are inadequate for non-shivering hypothermia [90]. Forced-air warming devices (e.g. Bair Hugger™ with underbody mattress and overlying blanket) are effective, safe, and well established. Non-invasive methods are rarely associated with excessive rewarming rates. Fluid and electrolyte disturbances, rhabdomyolysis, and renal failure are expected during rewarming and demand careful monitoring and management [35, 91, 92]. Arrhythmias may occur due to impaired conduction at low myocardial temperatures. Hypotension related to rewarming should be treated in accordance with local protocols. Transfer to an ECLS center should be considered for hypothermic patients (core temperature <30°C) not in CA with preserved cardiovascular function, or those for whom other rewarming methods have not been effective [7, 35, 93]. The role of ECLS rewarming in hypothermic patients not in CA is however still to be determined [8, 93].

Pulmonary edema, either from aspiration of snow particles or negative pressure pulmonary edema, is a recognized complication of avalanche burial [80, 83, 84]. It may also occur as a complication of rewarming [91].

The prognosis is generally favourable following treatment, usually with non-invasive ventilation [4].

### Patients in cardiac arrest at hospital admission

The hospital management for patients in CA at hospital admission should focus on the detection of candidates for ECLS rewarming. An important step of the in-hospital triage is to obtain the most accurate possible information regarding the mechanism and circumstances of CA, to determine if hypothermia may have been the cause. Accurate burial time and CPR time should be established through dispatch center and emergency teams prior to patient arrival at the hospital.

**Fig. 4:**
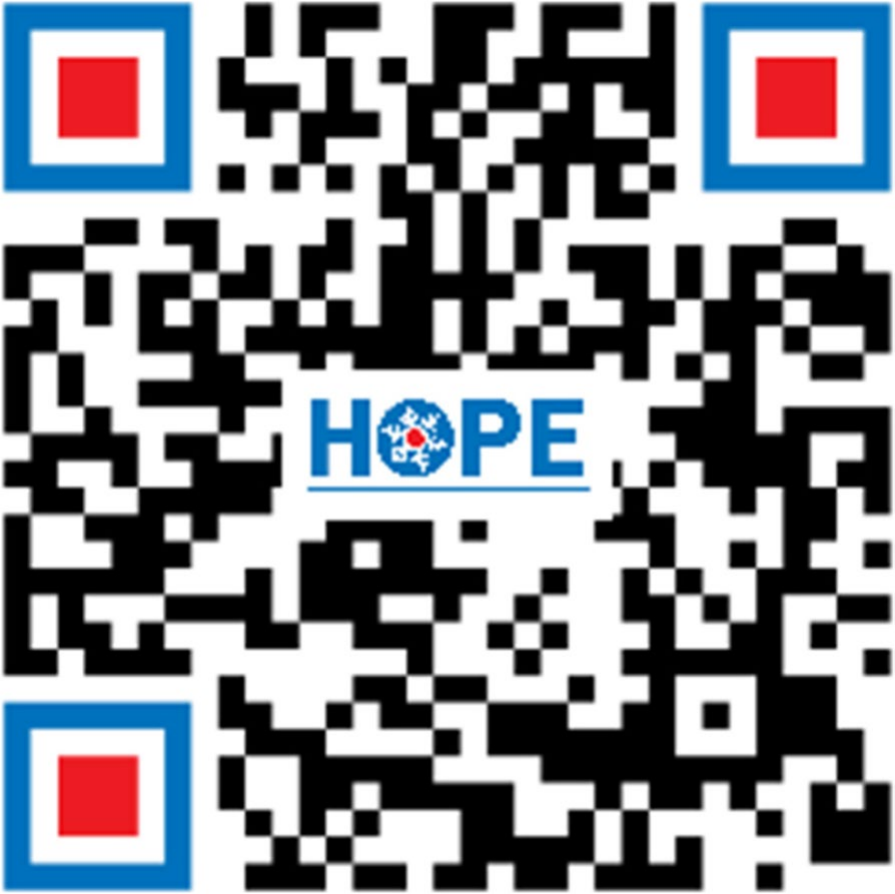
The Hypothermia Outcome Prediction (HOPE) score QR-code

Asphyxia or trauma are likely to be the cause of CA for patients with a core temperature ≥30°C. Critically buried patients with witnessed CA at extrication following long burial should be considered to have hypothermic rather than asphyxial CA [4]. For avalanche patients with a core temperature <30°C presenting with CA after critical burial, survival prediction and ECLS rewarming indication should rely on the Hypothermia Outcome Prediction after ECLS (HOPE) score [2, 4, 7, 10]. This externally validated score uses six variables available at hospital admission: sex, age, mechanism of hypothermia (presence or absence of asphyxia), duration of cardiopulmonary resuscitation (from beginning of CPR to anticipated initiation of ECLS), serum potassium and admission temperature. These variables provide individualized probabilities of survival to hospital discharge [81]. The HOPE score outperforms serum potassium alone as a triage tool to determine whether to rewarm hypothermic patients in CA, using a cut off of 10% [4, 7]. In a study of 46 patients with hypothermic CA (13 avalanche patients), HOPE-guided triage enabled better selection for ECLS rewarming than potassium alone [94]. An online calculator is freely available (https://www.hypothermiascore.org, Fig. 4). Asystole, long no-flow and low-flow times, unwitnessed CA, and advanced age are not contraindications for ECLS in hypothermic CA patients [8]. End-tidal CO_2_ should not be used to guide prognostication in hypothermic CA patients, including avalanche cases [95]. Trauma is frequently associated with avalanche accidents, but even severe trauma does not preclude favourable outcomes in hypothermic CA patients [96]. In the HOPE derivation and validation cohorts, critical burial of the head under the snow was classified as an asphyxia mechanism and associated with lower survival probability [10, 81]. However, some patients may survive the asphyxia and subsequently develop deep hypothermia, leading to CA [47, 78, 96–98]. This may occur in the presence of an air pocket [15], which is rarely reported [99] and was not included in the HOPE derivation study. It is therefore recommended that the “non-asphyxial” scenario should be considered to calculate the HOPE score if there is any suspicion that the patient may have survived asphyxia despite full burial [4, 7, 78]. This approach avoids underestimating survival probability and maximizes the chances of including patients in ECLS rewarming strategies [4, 78].

Since all prognostic tools have inherent limitations, the decision to rewarm avalanche patients with hypothermic CA ultimately rests on the treating team, who must integrate survival estimates with clinical judgment, the overall context, and the predicted survival probabilities provided by the HOPE score [4, 98]. If indicated, rewarming should be performed with veno-arterial extracorporeal membrane oxygenation (VA-ECMO). Transoesophageal echocardiography may help to ensure proper positioning of the ECMO cannulas [100]. The target rewarming rate is up to 5°C/hour and minimal or no anticoagulation should be used if there are signs of trauma [8]. ROSC may occur spontaneously (usually at temperatures of about 28–30°C) or following a shockable rhythm and successful defibrillation [100]. Serial echocardiography are proposed to assist weaning from ECLS [8].

## Outcome

The prognosis for avalanche buried patients without CA depends on the extend of potential trauma and the presence of posttraumatic stress disorder symptoms [91, 101].

**Fig. 5:**
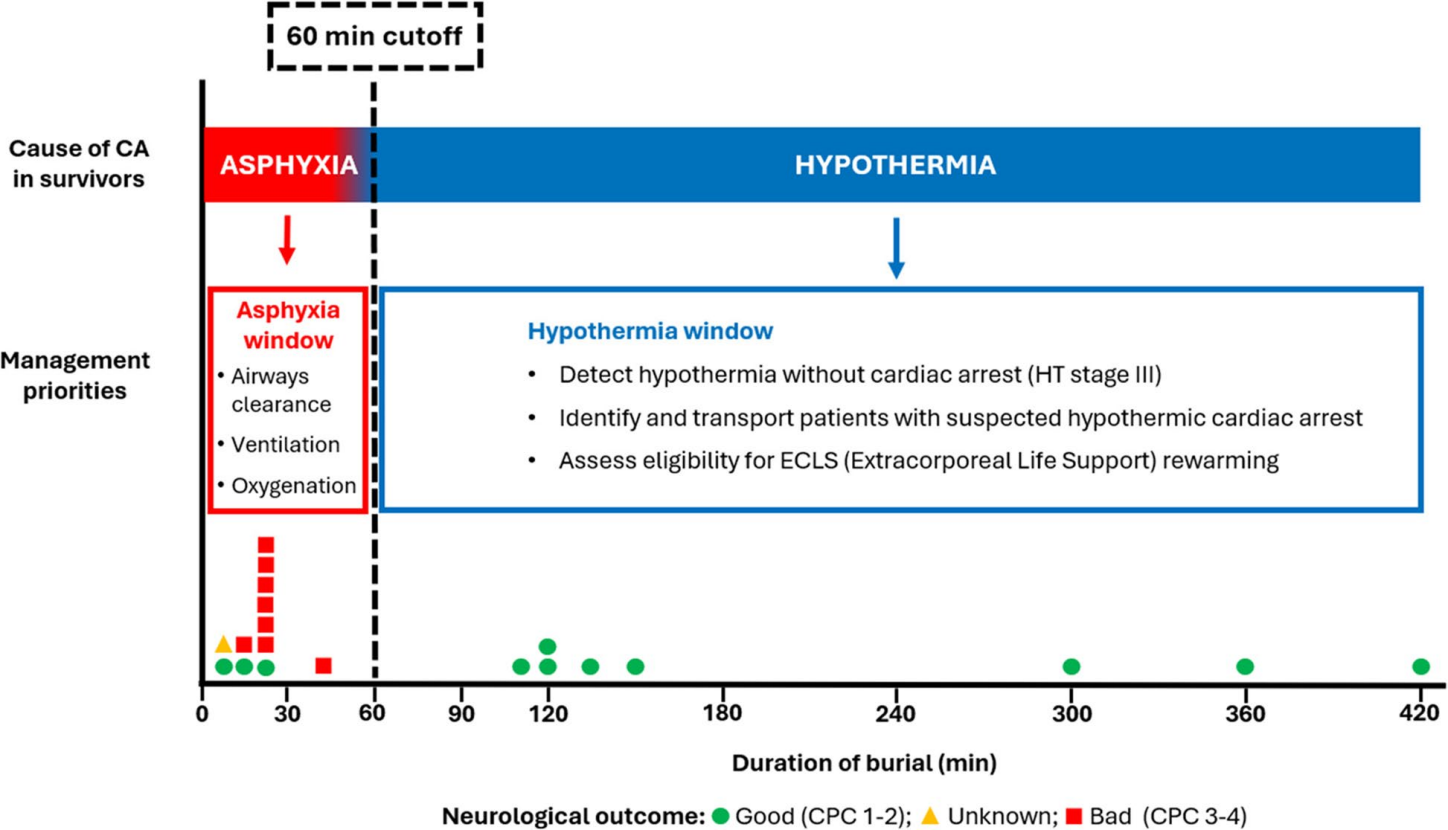
Principles of management of avalanche patients sustaining cardiac arrest (CA) from critical burial. Duration of burial and neurological outcome to hospital discharge of 20 survivors of avalanche burial with CA at extrication for whom burial duration was reported (data from Pasquier et al. 2023 [4]). «Asphyxia window» In case of short (≤60 min) burial, management should focus on rapid extrication, airway clearance and ventilation with oxygen as soon as practically feasible. Hypothermia is unlikely to be the cause of CA in case of short (≤ 60 min) burial unless in exceptional circumstances. «Hypothermia window» In case of long (>60 min) burial, hypothermia may be the cause of CA in patients who initially survived the «asphyxia» phase. Unless presence of lethal injuries or if hypothermic CA can be excluded by measuring core temperature (≥30°C), patients in CA and with patent airway should be transported with ongoing CPR to a hospital with ECLS. Indication for ECLS rewarming at hospital should include estimation of the survival probabilities using the HOPE score. CA: cardiac arrest; CPC: cerebral performance category; CPR: cardiopulmonary resuscitation; ECLS: extracorporeal life support; HOPE: Hypothermia. Outcome Prediction score after ECLS; HT: hypothermia.

Prognosis for out-of-hospital CA following avalanche burial remains poor [102, 103], and is significantly lower compared to other forms of hypothermic CA not associated with avalanche burial (12% vs. 23–100%) [10, 16, 17, 104]. Two main survivor profiles can be distinguished in avalanche-induced CA (Fig. 5). The first includes patients with asphyxic CA after short burial who typically achieve ROSC at the scene but often survive with poor neurological outcomes [7, 33, 102, 105]. The second group consists of patients with hypothermic CA after prolonged burial. They should be transported with ongoing CPR, undergo ECLS rewarming, and, when survival occurs, usually demonstrate excellent neurological recovery [7, 11, 33, 106, 107]. In contrast, the prognosis for unwitnessed asystolic hypothermic CA is extremely poor [51]. Among avalanche CA survivors, 83% of those with hypothermic CA achieved a Cerebral Performance Category (CPC) score of 1–2, compared with only 37% of survivors from non-hypothermic CA [4].

Evidence suggests that avalanche patients who may be candidates for rewarming are often undertreated. Among 140 patients with critical burial >60 min but <24 h, 19% survived, none of whom had CA at the scene [98]. In HEMS studies, about half of CA patients were declared dead on-scene, with or without attempted resuscitation [78, 103, 105]. Only a minority, including those with long burial and resuscitation provided on-scene, were transported to hospital [105, 108]. Improved detection strategies are therefore required to ensure that potentially salvageable hypothermic CA patients receive ECLS rewarming, when indicated. Thus, although overall survival is rare, long burial followed by hypothermic CA offers the best chances for meaningful recovery, and the transport of patients with patent airways should be favoured with the use of the Avalanche Victim Resuscitation Checklist [4, 44, 51].

### The avalanche patient as an organ donor

Even in the absence of survival, avalanche patients may contribute as organ donors. In one retrospective study, eight donors successfully provided 33 organs (13 kidneys, 6 livers, 3 pancreases, 5 hearts, 4 lungs) [109]. Donors typically had critical burials, achieved ROSC before hospital admission and did not undergo ECLS rewarming, as their course was not consistent with hypothermic CA. In another series of 66 avalanche patients who did not survive CA, seven became organ donors: five after ROSC and two after ECLS rewarming [103]. These findings highlight the importance of considering organ donation in the management pathway of avalanche patients with CA.

## Mass casualty incidents

A MCI overwhelms avalanche responders, where resources may not be sufficient to meet the demands of all patients, requiring a shift to maximize survival of the whole. Accidents involving multiple casualties comprised between 10 to 32% of operations [41–43]. In a MCI, the priority is to maximize the number of lives saved, placing greater emphasis on patients most likely to survive while minimizing time spent resuscitating those less likely to survive. Remote triage has been described during the MCI search phase and prioritizes shallow burials with higher survival potential over deeper ones due to excavation time and limited resources. Avalanche Survival Optimizing Rescue Triage (AvSORT) is the first algorithm designed specifically for avalanche MCIs, adapting already existing triage tools to austere mountain settings [110]. The system emphasized simple assessments, hemorrhage control and airway clearance while discouraging prolonged CPR if other subjects remained buried. Monte Carlo modeling for two buried subjects with one rescuer, concluded that limiting CPR to less than 20 min can maximize overall survival when another subject remains buried [111]. This has not been externally validated yet and may be challenged as the mathematical model includes simulations using data from CA from other causes than avalanches [112].

The International Commission for Alpine Rescue - MedCom guidelines for multi-casualty incidents in mountain rescue recommend extrication as the first priority for short burials, focusing on patients with signs of life until resources allow for additional treatment [113]. For burials between 35 and 60 min, CPR should be started on patients in CA only if resources are available. For burials over 60 min, CPR should only be initiated if the airway is patent. They support the use of checklists for improved triage and treatment [4, 6]. AvSORT II updated the original algorithm by incorporating terrain, burial depth, and extrication triage, allowing provisional black designations for low-survivability situations [114]. It considered burials deeper than 2 m as low priority, and rescuers should move on to the next subject. Similarly, the updated Wilderness Medical Society guidelines on avalanche management emphasized prioritization of patients based on terrain, vegetation, snowpack, avalanche risk, and burial depth during the search phase of MCI [2]. While they did not define new cut-offs for depth of burial nor CPR duration, they acknowledged AvaLife’s thresholds of only excavating subjects if <150 cm burial depth and limiting CPR to 6 min in resource-limited multiple burial scenarios when buried subjects remain [115].

Evacuation decisions in avalanche MCI are based on the patient’s medical condition (i.e. vital signs, co-morbidities, diagnosis and injury severity scores), the availability of helicopter and ground transport, and the nearest appropriate hospital. If there are multiple hypothermic CA patients, transport decisions can be based on the HOPE score in the nearest hospital [10, 81]. Hypothermic CA patients with a HOPE score ≥10% should be transferred to an ECLS center by HEMS [2, 4, 7, 81].

## Current realities and future development models

Out-of-hospital management of avalanche emergencies varies substantially worldwide, shaped by geography, rescue logistics, and organizational models of EMS [116]. In most European countries, a dense network of SAR and HEMS enables rapid responses to avalanche accidents, often within minutes. Ground and helicopter teams are usually staffed with ALS-trained healthcare providers. In contrast, prehospital care in North America, as well as in remote mountain regions of South America and Asia is typically provided by BLS-trained emergency medical technicians and ALS-trained paramedics, with physician-staffed helicopters being uncommon [116].

Although rescue times of critically buried avalanche subjects have been markedly reduced and survival improved since 1981 [14, 26–28, 37, 39, 117–119], studies and clinical practice highlight areas for improvement. In one study, airway assessment was documented in only 50% of avalanche patients, suggesting lower-than-expected compliance [99]. Survival of avalanche patients depends foremost on burial duration and the speed of extrication [5, 119]. Interventions that accelerate search and rescue are particularly effective within the first 30 min of burial. Widespread bystander training in BLS is vital, as well as the usage of safety device that can reduce the number of burials or prolong the survival when critically buried [26–28, 37, 39]. Drones may become increasingly useful for the remote detection of buried subjects, particularly in MCIs [120, 121], and their role is likely to expand as technology advances. Future advances may include further development and validation of current MCI algorithm, implementation of existing and developing technologies and studying the effects of climate change [18, 42, 43].

Implementation of knowledge and adherence to recommendations by SAR team and healthcare providers remains inconsistent, often due to limited awareness of key survival determinants [44, 54, 122]. Strengthening education, training, and implementation of checklist and SOPs across all levels of care, both prehospital and in-hospital, could further improve outcomes for avalanche patients.

## Conclusions

Avalanche fatalities result from asphyxia, trauma, and hypothermia. Prehospital and in-hospital care of an avalanche accident requires medical, technical, logistical, and organizational competencies. Survival for critically buried subjects relies on the speed of extrication, absence of trauma, presence of a patent airway and air pocket, immediate field treatment, and rapid transport to the most appropriate hospital. Survival may be improved with helicopter EMS, advancements in safety and rescue equipment technology, early deployment of professional SAR teams with checklists and SOPs.

## Supplementary Information

Supplementary Material 1. Supplemental Fig. S1. Avalanche Victim Resuscitation Checklist for clinical decision support and documentation in the field. (modified from Pasquier et al., 2023 with permission [4]).

## Data Availability

This is a review article, which relies on previous publications. Therefore, no new data have been uploaded to a repository.

## References

[1] Techel F, Jarry F, Kronthaler G, Mitterer S, Nairz P, Pavšek M, et al. Avalanche fatalities in the European Alps: long-term trends and statistics. Geogr Helv. 2016;71:147–59.

[2] Van Tilburg C, Paal P, Strapazzon G, Grissom CK, Haegeli P, Hölzl N, et al. Wilderness medical society clinical practice guidelines for prevention and management of avalanche and nonavalanche snow burial accidents: 2024 update. Wilderness Environ Med. 2024;35(1_suppl):20S-44S. 37945433 10.1016/j.wem.2023.05.014

[3] Strapazzon G, Taboni A, Dietrichs ES, Luks AM, Brugger H. Avalanche burial pathophysiology - a unique combination of hypoxia, hypercapnia and hypothermia. J Physiol. 2024;602(21):5785–800. 39073871 10.1113/JP284607

[4] Pasquier M, Strapazzon G, Kottmann A, Paal P, Zafren K, Oshiro K, et al. On-site treatment of avalanche victims: Scoping review and 2023 recommendations of the international commission for mountain emergency medicine (ICAR MedCom). Resuscitation. 2023;184:109708. 36709825 10.1016/j.resuscitation.2023.109708

[5] Strapazzon G, Brugger H. On-site treatment of snow avalanche victims: from bench to mountainside. High Alt Med Biol. 2018;19(4):307–15. 30183350 10.1089/ham.2018.0036

[6] Kottmann A, Blancher M, Spichiger T, Elsensohn F, Létang D, Boyd J, et al. The avalanche victim resuscitation checklist, a new concept for the management of avalanche victims. Resuscitation. 2015;91:e7-8. 25796998 10.1016/j.resuscitation.2015.03.009

[7] Lott C, Karageorgos V, Abelairas-Gomez C, Alfonzo A, Bierens J, Cantellow S, et al. European Resuscitation Council Guidelines 2025 Special Circumstances in Resuscitation. Resuscitation. 2025;215(Suppl 1):110753. 41117569 10.1016/j.resuscitation.2025.110753

[8] Cools E, Swol J, Wanscher M, Brugger H, Pasquier M, McIntosh S, et al. ELSO 2025 Narrative Guideline on the Use of ECMO for Accidental Hypothermia. ASAIO J. 2025. Epub ahead of print. 10.1097/MAT.0000000000002557. 10.1097/MAT.0000000000002557 41128452

[9] Strapazzon G, Forti A, Rauch S, Brugger H. The integration of prehospital standard operating procedures and in-hospital HOPE score for management of hypothermic patients in cardiac arrest. Resuscitation. 2019;141:212–3. 31238152 10.1016/j.resuscitation.2019.05.041

[10] Pasquier M, Hugli O, Paal P, Darocha T, Blancher M, Husby P, et al. Hypothermia outcome prediction after extracorporeal life support for hypothermic cardiac arrest patients: The HOPE score. Resuscitation. 2018;126:58–64. 29481910 10.1016/j.resuscitation.2018.02.026

[11] Brugger H, Bouzat P, Pasquier M, Mair P, Fieler J, Darocha T, et al. Cut-off values of serum potassium and core temperature at hospital admission for extracorporeal rewarming of avalanche victims in cardiac arrest: A retrospective multi-centre study. Resuscitation. 2019;139:222–9. 31022496 10.1016/j.resuscitation.2019.04.025

[12] Brugger H, Durrer B, Adler-Kastner L, Falk M, Tschirky F. Field management of avalanche victims. Resuscitation. 2001;51(1):7–15. 11719168 10.1016/s0300-9572(01)00383-5

[13] Falk M, Brugger H, Adler-Kastner L. Avalanche survival chances. Nature. 1994;368(6466):21. 7969398 10.1038/368021a0

[14] Rauch S, Brugger H, Falk M, Zweifel B, Strapazzon G, Albrecht R, et al. Avalanche survival rates in Switzerland, 1981–2020. JAMA Netw Open. 2024;7(9):e2435253. 39320893 10.1001/jamanetworkopen.2024.35253 PMC11425148

[15] Procter E, Strapazzon G, Dal Cappello T, et al. Burial duration, depth and air pocket explain avalanche survival patterns in Austria and Switzerland. Resuscitation. 2016;105:173–6. 27312137 10.1016/j.resuscitation.2016.06.001

[16] Haegeli P, Falk M, Brugger H, Etter HJ, Boyd J. Comparison of avalanche survival patterns in Canada and Switzerland. CMAJ. 2011;183(7):789–95. 21422139 10.1503/cmaj.101435 PMC3080528

[17] Strapazzon G, Paal P, Schweizer J, et al. Effects of snow properties on humans breathing into an artificial air pocket - an experimental field study. Sci Rep. 2017;7(1):17675. 29247235 10.1038/s41598-017-17960-4 PMC5732296

[18] Strapazzon G, Schweizer J, Chiambretti I, Brodmann Maeder M, Brugger H, Zafren K. Effects of climate change on avalanche accidents and survival. Front Physiol. 2021;12:639433. 33912070 10.3389/fphys.2021.639433 PMC8072472

[19] Paal P, Strapazzon G, Braun P, Ellmauer PP, Schroeder DC, Sumann G, et al. Factors affecting survival from avalanche burial–a randomised prospective porcine pilot study. Resuscitation. 2013;84(2):239–43. 22771873 10.1016/j.resuscitation.2012.06.019

[20] Boyd J, Brugger H, Shuster M. Prognostic factors in avalanche resuscitation: a systematic review. Resuscitation. 2010;81(6):645–52. 20371145 10.1016/j.resuscitation.2010.01.037

[21] Heschl S, Paal P, Farzi S, Toller W. Electrical cardiac activity in an avalanche victim dying of asphyxia. Resuscitation. 2013;84(11):e143-4. 23891774 10.1016/j.resuscitation.2013.07.014

[22] Wik L, Brattebø G, Østerås Ø, Assmus J, Irusta U, Aramendi E, et al. Physiological effects of providing supplemental air for avalanche victims. A randomised trial. Resuscitation. 2022;172:38–46. 35063621 10.1016/j.resuscitation.2022.01.007

[23] Strapazzon G, Gatterer H, Falla M, Dal Cappello T, Malacrida S, Turner R, et al. Hypoxia and hypercapnia effects on cerebral oxygen saturation in avalanche burial: a pilot human experimental study. Resuscitation. 2021;158:175–82. 33249253 10.1016/j.resuscitation.2020.11.023

[24] Brugger H, Sumann G, Meister R, et al. Hypoxia and hypercapnia during respiration into an artificial air pocket in snow: implications for avalanche survival. Resuscitation. 2003;58(1):81–8. 12867313 10.1016/s0300-9572(03)00113-8

[25] McIntosh SE, Little CE, Seibert TD, Polukoff NE, Grissom CK. Avalanche airbag post-burial active deflation - The ability to create an air pocket to delay asphyxiation and prolong survival. Resuscitation. 2020;146:155–60. 31812665 10.1016/j.resuscitation.2019.11.023

[26] Eisendle F, Roveri G, Rauch S, Thomassen Ø, Dal Cappello T, Assmus J, Malacrida S, Kammerer T, Schweizer J, Borasio N, Dörck V, Falk M, et al. Respiratory Gas Shifts to Delay Asphyxiation in Critical Avalanche Burial: A Randomized Clinical Trial. JAMA. 2025:e2516837. Epub ahead of print. 10.1001/jama.2025.16837. 10.1001/jama.2025.16837 PMC12509078 41060661

[27] Strapazzon G, Rauch S, Malacrida S, Dal Cappello T, Governo E, Catuzzo B, et al. Comparative effectiveness of an artificial air pocket device to delay asphyxiation in supine individuals critically buried in avalanche debris. JAMA Netw Open. 2023;6(5):e2313376. 37184835 10.1001/jamanetworkopen.2023.13376 PMC12578492

[28] Grissom CK, Radwin MI, Harmston CH, Hirshberg EL, Crowley TJ. Respiration during snow burial using an artificial air pocket. JAMA. 2000;283(17):2266–71. 10807386 10.1001/jama.283.17.2266

[29] Grissom CK, McAlpine JC, Harmston CH, Radwin MI, Giesbrecht GG, Scholand MB, et al. Hypercapnia effect on core cooling and shivering threshold during snow burial. Aviat Space Environ Med. 2008;79(8):735–42. 18717110 10.3357/asem.2261.2008

[30] Brugger H, Procter E, Rauch S, Strapazzon G. Cooling rate for triage decisions should exclude post-extrication cooling in avalanche victims. Resuscitation. 2015;94:e3. 26159611 10.1016/j.resuscitation.2015.06.020

[31] Mittermair C, Foidl E, Wallner B, Brugger H, Paal P. Extreme cooling rates in avalanche victims: case report and narrative review. High Alt Med Biol. 2021;22(2):235–40. 33761270 10.1089/ham.2020.0222

[32] Mair P, Brugger H, Mair B, Moroder L, Ruttmann E. Is extracorporeal rewarming indicated in avalanche victims with unwitnessed hypothermic cardiorespiratory arrest? High Alt Med Biol. 2014;15(4):500–3. 25531463 10.1089/ham.2014.1066

[33] Boue Y, Payen JF, Brun J, et al. Survival after avalanche-induced cardiac arrest. Resuscitation. 2014;85(9):1192–6. 24971508 10.1016/j.resuscitation.2014.06.015

[34] Hilmo J, Naesheim T, Gilbert M. “Nobody is dead until warm and dead”: Prolonged resuscitation is warranted in arrested hypothermic victims also in remote areas - A retrospective study from northern Norway. Resuscitation. 2014;85(9):1204–11. 24882104 10.1016/j.resuscitation.2014.04.029

[35] Paal P, Gordon L, Strapazzon G, BrodmannMaeder M, Putzer G, Walpoth B, et al. Accidental hypothermia-an update: The content of this review is endorsed by the International Commission for Mountain Emergency Medicine (ICAR MEDCOM). Scand J Trauma Resusc Emerg Med. 2016;24(1):111. 27633781 10.1186/s13049-016-0303-7 PMC5025630

[36] Milani M, Roveri G, Falla M, Dal Cappello T, Strapazzon G. Occupational accidents among search and rescue providers during mountain rescue operations and training events. Ann Emerg Med. 2023;81(6):699–705. 36669910 10.1016/j.annemergmed.2022.12.015

[37] Procter E, Strapazzon G, Dal Cappello T, Castlunger L, Staffler HP, Brugger H. Adherence of backcountry winter recreationists to avalanche prevention and safety practices in northern Italy. Scand J Med Sci Sports. 2014;24(5):823–9. 23815413 10.1111/sms.12094

[38] Brugger H, Etter HJ, Zweifel B, et al. The impact of avalanche rescue devices on survival. Resuscitation. 2007;75(3):476–83. 17689170 10.1016/j.resuscitation.2007.06.002

[39] Haegeli P, Falk M, Procter E, et al. The effectiveness of avalanche airbags. Resuscitation. 2014;85(9):1197–203. 24909367 10.1016/j.resuscitation.2014.05.025

[40] Pietsch U, Moeckel J, Koppenberg J, Josi D, Jungwirth A, Hautz WE, et al. Stability of Drugs Stored in Helicopters for Use by Emergency Medical Services: A Prospective Observational Study. Ann Emerg Med. 2022;80(4):364–70. 35927113 10.1016/j.annemergmed.2022.05.038

[41] Mair P, Frimmel C, Vergeiner G, Hohlrieder M, Moroder L, Hoesl P, et al. Emergency medical helicopter operations for avalanche accidents. Resuscitation. 2013;84(4):492–5. 22986068 10.1016/j.resuscitation.2012.09.010

[42] Lunde A, Tellefsen C. Patient and rescuer safety: recommendations for dispatch and prioritization of rescue resources based on a retrospective study of Norwegian avalanche incidents 1996–2017. Scand J Trauma Resusc Emerg Med. 2019;27(1):5. 30642369 10.1186/s13049-019-0585-7 PMC6332597

[43] Kottmann A, Carron PN, Theiler L, Albrecht R, Tissi M, Pasquier M. Identification of the technical and medical requirements for HEMS avalanche rescue missions through a 15-year retrospective analysis in a HEMS in Switzerland: a necessary step for quality improvement. Scand J Trauma Resusc Emerg Med. 2018;26(1):54. 29973290 10.1186/s13049-018-0520-3 PMC6033290

[44] Trolliet M, Pasquier M, Blancher M, Albrecht R, Lovis A, Brugger H, et al. The impact of a dedicated checklist on the quality of onsite management of critically buried avalanche victims in cardiac arrest in a Swiss helicopter emergency medical service. Scand J Trauma Resusc Emerg Med. 2024;32(1):124. 39627884 10.1186/s13049-024-01300-3 PMC11613841

[45] Sumann G, Moens D, Brink B, BrodmannMaeder M, Greene M, Jacob M, et al. Multiple trauma management in mountain environments - a scoping review : Evidence based guidelines of the International Commission for Mountain Emergency Medicine (ICAR MedCom). Intended for physicians and other advanced life support personnel. Scand J Trauma Resusc Emerg Med. 2020;28(1):117. 33317595 10.1186/s13049-020-00790-1 PMC7737289

[46] Wallner B, Moroder L, Salchner H, Mair P, Wallner S, Putzer G, et al. CPR with restricted patient access using alternative rescuer positions: a randomised cross-over manikin study simulating the CPR scenario after avalanche burial. Scand J Trauma Resusc Emerg Med. 2021;29(1):129. 34481521 10.1186/s13049-021-00944-9 PMC8418718

[47] Strapazzon G, Beikircher W, Procter E, Brugger H. Electrical heart activity recorded during prolonged avalanche burial. Circulation. 2012;125(4):646–7. 22294709 10.1161/CIRCULATIONAHA.111.049783

[48] Pasquier M, Blancher M, Zen Ruffinen G, Hugli O. Does rescue collapse mandate a paradigm shift in the field management of avalanche victims? High Alt Med Biol. 2015;16(2):171–2. 25946378 10.1089/ham.2015.0012

[49] Strapazzon G, Procter E, Paal P, Brugger H. Pre-hospital core temperature measurement in accidental and therapeutic hypothermia. High Alt Med Biol. 2014;15(2):104–11. 24950388 10.1089/ham.2014.1008

[50] Pasquier M, Paal P, Kosinski S, Brown D, Podsiadlo P, Darocha T. Esophageal temperature measurement. N Engl J Med. 2020;383(16):e93. 33053286 10.1056/NEJMvcm1900481

[51] Althaus U, Aeberhard P, Schüpbach P, Nachbur BH, Mühlemann W. Management of profound accidental hypothermia with cardiorespiratory arrest. Ann Surg. 1982;195(4):492–5. 7065752 10.1097/00000658-198204000-00018 PMC1352533

[52] Lavonas EJ, Drennan IR, Gabrielli A, et al. Part 10: special circumstances of resuscitation: 2015 American Heart Association guidelines update for cardiopulmonary resuscitation and emergency cardiovascular care. Circulation. 2015;132(18 Suppl 2):S501-18. 26472998 10.1161/CIR.0000000000000264

[53] Morrison LJ, Verbeek PR, Zhan C, Kiss A, Allan KS. Validation of a universal prehospital termination of resuscitation clinical prediction rule for advanced and basic life support providers. Resuscitation. 2009;80(3):324–8. 19150167 10.1016/j.resuscitation.2008.11.014

[54] Kottmann A, Pasquier M, Strapazzon G, Zafren K, Ellerton J, Paal P. Quality indicators for avalanche victim management and rescue. Int J Environ Res Public Health. 2021;18(18):9570. 34574495 10.3390/ijerph18189570 PMC8464975

[55] McIntosh SE, Grissom CK, Olivares CR, Kim HS, Tremper B. Cause of death in avalanche fatalities. Wilderness Environ Med. 2007;18(4):293–7. 18076300 10.1580/07-WEME-OR-092R1.1

[56] Hohlrieder M, Brugger H, Schubert HM, Pavlic M, Ellerton J, Mair P. Pattern and severity of injury in avalanche victims. High Alt Med Biol. 2007;8(1):56–61. 17394418 10.1089/ham.2006.0815

[57] Lugnet V, McDonough M, Gordon L, Galindez M, Mena Reyes N, Sheets A, et al. Termination of cardiopulmonary resuscitation in mountain rescue: a scoping review and ICAR MedCom 2023 recommendations. High Alt Med Biol. 2023;24(4):274–86. 37733297 10.1089/ham.2023.0068

[58] Paal P, Milani M, Brown D, Boyd J, Ellerton J. Termination of cardiopulmonary resuscitation in mountain rescue. High Alt Med Biol. 2012;13(3):200–8. 22994520 10.1089/ham.2011.1096

[59] Schön CA, Gordon L, Hölzl N, Milani M, Paal P, Zafren K. Determination of death in mountain rescue: recommendations of the International Commission for Mountain Emergency Medicine (ICAR MedCom). Wilderness Environ Med. 2020;31(4):506–20. 33077333 10.1016/j.wem.2020.06.013

[60] Quinn RH, Williams J, Bennett BL, Stiller G, Islas AA, McCord S. Wilderness medical society practice guidelines for spine immobilization in the austere environment: 2014 update. Wilderness Environ Med. 2014;25(4 Suppl):S105-7. 25498256 10.1016/j.wem.2014.05.004

[61] Hawkins SC, Williams J, Bennett BL, Islas A, Kayser DW, Quinn R. Wilderness medical society clinical practice guidelines for spinal cord protection. Wilderness Environ Med. 2019;30(4S):S87–99. 31780084 10.1016/j.wem.2019.08.001

[62] Ong ME, Jaffey J, Stiell I, Nesbitt L, OPALS Study Group. Comparison of termination-of-resuscitation guidelines for basic life support: defibrillator providers in out-of-hospital cardiac arrest. Ann Emerg Med. 2006;47(4):337–43. 16546618 10.1016/j.annemergmed.2005.05.012

[63] Roveri G, Gamberini L, Borotto E, Eisendle F, Festi L, Brugger H, et al. Effect of cold environments on technical performance and perceived workload and stress during advanced medical procedures: a randomized controlled simulation study. Scand J Trauma Resusc Emerg Med. 2025;33(1):113. 40598316 10.1186/s13049-025-01373-8 PMC12211314

[64] Masè M, Micarelli A, Roveri G, Falla M, Dal Cappello T, van Veelen MJ, et al. Vital parameter monitoring in harsh environment by the MedSENS in-ear multisensor device. Sci Rep. 2024;14(1):19117. 39155284 10.1038/s41598-024-68936-0 PMC11330965

[65] Musi ME, Sheets A, Zafren K, Brugger H, Paal P, Hölzl N, et al. Clinical staging of accidental hypothermia: the revised Swiss system: recommendation of the International Commission for Mountain Emergency Medicine (ICAR MedCom). Resuscitation. 2021;162:182–7. 33675869 10.1016/j.resuscitation.2021.02.038

[66] Dow J, Giesbrecht GG, Danzl DF, Brugger H, Sagalyn EB, Walpoth B, et al. Wilderness Medical Society clinical practice guidelines for the out-of-hospital evaluation and treatment of accidental hypothermia: 2019 update. Wilderness Environ Med. 2019;30(4S):S47–69. 31740369 10.1016/j.wem.2019.10.002

[67] Pietsch U, Knapp J, Kreuzer O, Ney L, Strapazzon G, Lischke V, et al. Advanced airway management in hoist and longline operations in mountain HEMS - considerations in austere environments: a narrative review This review is endorsed by the International Commission for Mountain Emergency Medicine (ICAR MEDCOM). Scand J Trauma Resusc Emerg Med. 2018;26(1):23. 29615073 10.1186/s13049-018-0490-5 PMC5883516

[68] Boue Y, Lavolaine J, Bouzat P, Matraxia S, Chavanon O, Payen JF. Neurologic recovery from profound accidental hypothermia after 5 hours of cardiopulmonary resuscitation. Crit Care Med. 2014;42(2):e167-70. 10.1097/CCM.0b013e3182a643bc 24158171

[69] Brillhart A, Nowak C, Moore N, Silva E. Ski Patroller Manual Cardiopulmonary Resuscitation During Rescue Toboggan Transport: Three Vermont Skier Cases of Cardiac Arrest With Neurologically Intact Survival and Practical Suggestions for Implementation. Wilderness Environ Med. 2025:10806032251364148. Epub ahead of print. 10.1177/10806032251364148. 10.1177/10806032251364148 40831273

[70] Thomassen O, Skaiaa SC, Assmuss J, Østerås Ø, Heltne JK, Wik L, et al. Mountain rescue cardiopulmonary resuscitation: a comparison between manual and mechanical chest compressions during manikin cardio resuscitation. Emerg Med J. 2017;34(9):573–7. 28476730 10.1136/emermed-2016-206323

[71] Rupp SL, Overberger RC. Manual vs mechanical cardiopulmonary resuscitation for out-of-hospital cardiac arrest on a ski slope: a pilot study. Wilderness Environ Med. 2023;34(3):289–94. 37169609 10.1016/j.wem.2023.03.006

[72] Gordon L, Paal P, Ellerton JA, Brugger H, Peek GJ, Zafren K. Delayed and intermittent CPR for severe accidental hypothermia. Resuscitation. 2015;90:46–9. 25725297 10.1016/j.resuscitation.2015.02.017

[73] Gässler H, Kurka L, Rauch S, Seewald S, Kulla M, Fischer M. Mechanical chest compression devices under special circumstances. Resuscitation. 2022;179:183–8. 35738309 10.1016/j.resuscitation.2022.06.014

[74] Hollott J. Ventilatory choices for intubated patients during helicopter stretcher winching. Emerg Med Australas. 2017;29(6):692–6. 28845544 10.1111/1742-6723.12845

[75] Putzer G, Braun P, Zimmermann A, Pedross F, Strapazzon G, Brugger H, et al. LUCAS compared to manual cardiopulmonary resuscitation is more effective during helicopter rescue-a prospective, randomized, cross-over manikin study. Am J Emerg Med. 2012;31(2):384–9. 23000324 10.1016/j.ajem.2012.07.018

[76] Vögele A, van Veelen MJ, Dal Cappello T, Falla M, Nicoletto G, Dejaco A, et al. Effect of acute exposure to altitude on the quality of chest compression-only cardiopulmonary resuscitation in helicopter emergency medical services personnel: a randomized, controlled, single-blind crossover trial. J Am Heart Assoc. 2021;10(23):e021090. 34854317 10.1161/JAHA.121.021090 PMC9075389

[77] Callender N, Ellerton J, MacDonald JH. Physiological demands of mountain rescue work. Emerg Med J. 2012;29(9):753–7. 21960460 10.1136/emermed-2011-200485

[78] Rousson V, Hall N, Pasquier M. Recommendation on the Use of the HOPE Score at the Hospital for Outcome Prediction in Critically Buried Hypothermic Avalanche Victims Considered for ECLS Rewarming. Wilderness Environ Med. 2024;35(2):251–2. 38549350 10.1177/10806032241242084

[79] Meyer DE, Vincent LE, Fox EE, OʼKeeffe T, Inaba K, Bulger E, et al. Every minute counts: Time to delivery of initial massive transfusion cooler and its impact on mortality. J Trauma Acute Care Surg. 2017;83(1):19–24. 28452870 10.1097/TA.0000000000001531 PMC5526458

[80] Cohen JG, Grenier F, D’Alnoncourt S, Reymond E, Blancher M, Peoc’h M, et al. Asphyxia after complete avalanche burial: A new paradigm. Resuscitation. 2017;118:e1–2. 28746828 10.1016/j.resuscitation.2017.07.015

[81] Pasquier M, Rousson V, Darocha T, Bouzat P, Kosinski S, Sawamoto K, et al. Hypothermia outcome prediction after extracorporeal life support for hypothermic cardiac arrest patients: An external validation of the HOPE score. Resuscitation. 2019;139:321–8. 30940473 10.1016/j.resuscitation.2019.03.017

[82] Kosiński S, Podsiadło P, Pasquier M, Darocha T. Temperature measurement in severely traumatized patients. J Trauma Acute Care Surg. 2019;86(4):759. 30601457 10.1097/TA.0000000000002174

[83] Glisenti P, Rakusa J, Albrecht R, Luedi MM. Negative pressure pulmonary oedema with haemorrhage after 5-minute avalanche burial. Lancet. 2016;388(10057):2321–2. 27825509 10.1016/S0140-6736(16)31010-8

[84] Sumann G, Putzer G, Brugger H, Paal P. Pulmonary edema after complete avalanche burial. High Alt Med Biol. 2012;13(4):295–6. 23270451 10.1089/ham.2012.1072

[85] Hymczak H, Gołąb A, Mendrala K, Plicner D, Darocha T, Podsiadło P, et al. Core Temperature Measurement-Principles of Correct Measurement, Problems, and Complications. Int J Environ Res Public Health. 2021;18(20):10606. 10.3390/ijerph182010606 PMC8535559 34682351

[86] Matsukawa T, Sessler DI, Christensen R, Ozaki M, Schroeder M. Heat flow and distribution during epidural anesthesia. Anesthesiology. 1995;83(5):961–7. 7486181 10.1097/00000542-199511000-00008

[87] Vincent-Lambert C, Smith CM, Goldstein LN. Hypothermia in trauma patients arriving at an emergency department by ambulance in Johannesburg, South Africa: a prospective study. Pan Afr Med J. 2018;31:136. 31037196 10.11604/pamj.2018.31.136.13615 PMC6462367

[88] Mendrala K, Kosiński S, Podsiadło P, Pasquier M, Paal P, Mazur P, et al. The efficacy of renal replacement therapy for rewarming of patients in severe accidental hypothermia-systematic review of the literature. Int J Environ Res Public Health. 2021;18(18):9638. 34574562 10.3390/ijerph18189638 PMC8467292

[89] Kiridume K, Hifumi T, Kawakita K, Okazaki T, Hamaya H, Shinohara N, et al. Clinical experience with an active intravascular rewarming technique for near-severe hypothermia associated with traumatic injury. J Intensive Care. 2014;2(1):11. 25520827 10.1186/2052-0492-2-11 PMC4267585

[90] Mydske S, Helland AM, Borasio N, Brattebø G, Østerås Ø, Wiggen Ø, et al. Effect of active external rewarming on esophageal temperature in simulated prehospital accidental hypothermia: a randomized crossover trial. Scand J Trauma Resusc Emerg Med. 2025. 10.1186/s13049-025-01528-7. Epub ahead of print. 10.1186/s13049-025-01528-7 PMC12805701 41388306

[91] Strapazzon G, Nardin M, Zanon P, Kaufmann M, Kritzinger M, Brugger H. Respiratory failure and spontaneous hypoglycemia during noninvasive rewarming from 24.7°C (76.5°F) core body temperature after prolonged avalanche burial. Ann Emerg Med. 2012;60(2):193–6. 22153969 10.1016/j.annemergmed.2011.11.015

[92] Forti A, Brugnaro P, Rauch S, Crucitti M, Brugger H, Cipollotti G, et al. Hypothermic cardiac arrest with full neurologic recovery after approximately nine hours of cardiopulmonary resuscitation: management and possible complications. Ann Emerg Med. 2019;73(1):52–7. 30420231 10.1016/j.annemergmed.2018.09.018

[93] Mendrala K, Podsiadlo P, Darocha T. Extracorporeal life support in accidental hypothermia. JAMA. 2025;334(20):1846–7. 41060623 10.1001/jama.2025.14593

[94] Hall N, Metrailler-Mermoud J, Rousson V, Conforti C, Dupasquier A, Carron PN, et al. Use of the HOPE score to assess survival outcome of hypothermic cardiac arrest selected by ECLS rewarming. Scand J Trauma Resusc Emerg Med. 2025;33(1):132. 40721803 10.1186/s13049-025-01445-9 PMC12305985

[95] Darocha T, Debaty G, Ageron FX, Podsiadlo P, Hutin A, Hymczak H, et al. Hypothermia is associated with a low ETCO(2) and low pH-stat PaCO(2) in refractory cardiac arrest. Resuscitation. 2022;174:83–90. 35101599 10.1016/j.resuscitation.2022.01.022

[96] Maeder MB, Lischke V, Berner A, Reisten O, Pietsch U, Pasquier M. A patient with polytrauma, hypothermia and cardiac arrest after delayed mountain rescue. CMAJ. 2018;190(42):E1263. 30348744 10.1503/cmaj.70338 PMC6199164

[97] Bracco C, Strapazzon G, Sciolla A, Dupuis A, Lauria G, Fenoglio L. A critically prolonged avalanche burial with recorded cardiac electrical activity and complete recovery - a case report. Scand J Trauma Resusc Emerg Med. 2024;32(1):61. 38961504 10.1186/s13049-024-01230-0 PMC11223440

[98] Eidenbenz D, Techel F, Kottmann A, Rousson V, Carron PN, Albrecht R, et al. Survival probability in avalanche victims with long burial (>/=60 min): A retrospective study. Resuscitation. 2021;166:93–100. 34107337 10.1016/j.resuscitation.2021.05.030

[99] Eisendle F, Rauch S, Wallner B, Brugger H, Strapazzon G. Prevalence of airway patency and air pocket in critically buried avalanche victims - a scoping review. Scand J Trauma Resusc Emerg Med. 2024;32(1):34. 38654361 10.1186/s13049-024-01205-1 PMC11040957

[100] Swol J, Darocha T, Paal P, Brugger H, Podsiadło P, Kosiński S, et al. Extracorporeal Life Support in Accidental Hypothermia with Cardiac Arrest-A Narrative Review. ASAIO J. 2022;68(2):153–62. 34261875 10.1097/MAT.0000000000001518 PMC8797003

[101] Leonard C, Charriau-Perret A, Debaty G, Belle L, Ricard C, Sanchez C, et al. Survivors of avalanche accidents: posttraumatic stress disorder symptoms and quality of life: a multicentre study. Scand J Trauma Resusc Emerg Med. 2021;29(1):96. 34281606 10.1186/s13049-021-00912-3 PMC8287800

[102] Knapp J, Hoftmann D, Albrecht R, Straumann S, Pasquier M, Pietsch U. Management and outcome of patients with cardiac arrest after avalanche accidents in the Swiss Alps: a retrospective analysis. Resusc Plus. 2025;22:100922. 40161289 10.1016/j.resplu.2025.100922 PMC11951987

[103] Metrailler-Mermoud J, Hugli O, Carron PN, Kottmann A, Frochaux V, Zen-Ruffinen G, et al. Avalanche victims in cardiac arrest are unlikely to survive despite adherence to medical guidelines. Resuscitation. 2019;141:35–43. 31185258 10.1016/j.resuscitation.2019.05.037

[104] Wanscher M, Agersnap L, Ravn J, Yndgaard S, Nielsen JF, Danielsen ER, et al. Outcome of accidental hypothermia with or without circulatory arrest: experience from the Danish Præstø Fjord boating accident. Resuscitation. 2012;83:1078–84. 22634431 10.1016/j.resuscitation.2012.05.009

[105] Moroder L, Mair B, Brugger H, Voelckel W, Mair P. Outcome of avalanche victims with out-of-hospital cardiac arrest. Resuscitation. 2015;89:114–8. 25625778 10.1016/j.resuscitation.2015.01.019

[106] Oberhammer R, Beikircher W, Hormann C, Lorenz I, Pycha R, Adler-Kastner L, et al. Full recovery of an avalanche victim with profound hypothermia and prolonged cardiac arrest treated by extracorporeal re-warming. Resuscitation. 2008;76(3):474–80. 17988783 10.1016/j.resuscitation.2007.09.004

[107] Kosinski S, Darocha T, Jarosz A, Migiel L, Zelias A, Marcinkowski W, et al. The longest persisting ventricular fibrillation with an excellent outcome - 6h 45min cardiac arrest. Resuscitation. 2016;105:e21-2. 27283064 10.1016/j.resuscitation.2016.05.022

[108] Strapazzon G, Plankensteiner J, Mair P, Ruttmann E, Dal Cappello T, Procter E, et al. Prehospital management and outcome of avalanche patients with out-of-hospital cardiac arrest: a retrospective study in Tyrol. Austria European journal of emergency medicine. 2017;24(6):398–403. 26990382 10.1097/MEJ.0000000000000390

[109] Gasteiger L, Putzer G, Unterpertinger R, Cardini B, Schneeberger S, Eschertzhuber S, et al. Solid organ donation from brain-dead donors with cardiorespiratory arrest after snow avalanche burial: a retrospective single-center study. Transplantation. 2022;106(3):584–7. 33859150 10.1097/TP.0000000000003785

[110] Bogle LB, Boyd JJ, McLaughlin KA. Triaging multiple victims in an avalanche setting: the Avalanche survival optimizing rescue triage algorithmic approach. Wilderness Environ Med. 2010;21(1):28–34. 20591351 10.1016/j.wem.2009.12.002

[111] Reiweger I, Genswein M, Paal P, Schweizer J. A concept for optimizing avalanche rescue strategies using a Monte Carlo simulation approach. PLoS One. 2017;12(5):e0175877. 28467434 10.1371/journal.pone.0175877 PMC5414947

[112] Reynolds JC, Frisch A, Rittenberger JC, Callaway CW. Duration of resuscitation efforts and functional outcome after out-of-hospital cardiac arrest: when should we change to novel therapies? Circulation. 2013;128(23):2488–94. 24243885 10.1161/CIRCULATIONAHA.113.002408 PMC4004337

[113] Blancher M, Albasini F, Elsensohn F, Zafren K, Hölzl N, McLaughlin K, et al. Management of multi-casualty incidents in mountain rescue: evidence-based guidelines of the International Commission for Mountain Emergency Medicine (ICAR MEDCOM). High Alt Med Biol. 2018;19(2):131–40. 29446647 10.1089/ham.2017.0143 PMC6014052

[114] Inniss M. AvSORT II: multi-casualty avalanche triage algorithm. The Avalanche Journal. 2020;125:28–30.

[115] Genswein M, Macias D, McIntosh S, Reiweger I, Hetland A, Paal P. AvaLife-a new multi-disciplinary approach supported by accident and field test data to optimize survival chances in rescue and first aid of avalanche patients. Int J Environ Res Public Health. 2022;19(9):5257. 35564653 10.3390/ijerph19095257 PMC9104102

[116] Brugger H, Elsensohn F, Syme D, Sumann G, Falk M. A survey of emergency medical services in mountain areas of Europe and North America: official recommendations of the International Commission for Mountain Emergency Medicine (ICAR Medcom). High Alt Med Biol. 2005;6(3):226–37. 16185140 10.1089/ham.2005.6.226

[117] Brugger H, Etter HJ, Zweifel B, Mair P, Hohlrieder M, Ellerton J, et al. The impact of avalanche rescue devices on survival. Resuscitation. 2007;75(3):476–83. 17689170 10.1016/j.resuscitation.2007.06.002

[118] Wallner B, Woyke S, Winkler M, Caramazza F, Regli IB, Putzer G, et al. Avalanche transceiver search times during avalanche companion rescue - a prospective randomized single-blinded cross-over simulation study. Resusc Plus. 2025;26:101065. 40927180 10.1016/j.resplu.2025.101065 PMC12415072

[119] Wallner B, Moroder L, Brandt A, Mair P, Erhart S, Bachler M, et al. Extrication times during avalanche companion rescue: a randomized single-blinded manikin study. High Alt Med Biol. 2019;20(3):245–50. 31264903 10.1089/ham.2019.0021

[120] Abrahamsen HB. A remotely piloted aircraft system in major incident management: concept and pilot, feasibility study. BMC Emerg Med. 2015;15:12. 26054527 10.1186/s12873-015-0036-3 PMC4460697

[121] van Veelen MJ, Roveri G, Voegele A, Dal Cappello T, Masè M, Falla M, et al. Drones reduce the treatment-free interval in search and rescue operations with telemedical support - A randomized controlled trial. Am J Emerg Med. 2023;66:40–4. 36680868 10.1016/j.ajem.2023.01.020

[122] Strapazzon G, Migliaccio D, Fontana D, Stawinoga AE, Milani M, Brugger H. Knowledge of the avalanche victim resuscitation checklist and utility of a standardized lecture in Italy. Wilderness Environ Med. 2018;29(1):56–60. 29074075 10.1016/j.wem.2017.08.007

