# Supplementary material for: Emergency care for avalanche buried patients - a narrative review
Source: Scand J Trauma Resusc Emerg Med. 2026 Feb 20;34:63. doi: 10.1186/s13049-026-01543-2 (PMC13032496; doi:10.1186/s13049-026-01543-2)
Supplement: Supplementary file 1 — Supplementary Material 1. Supplemental Fig. S1. Avalanche Victim Resuscitation Checklist for clinical decision support and documentation in the field. (modified from Pasquier et al., 2023 with permission [4]). [file 13049_2026_1543_MOESM1_ESM.pdf]

**Patient ID:**



©ICAR MEDCOM, Jan 2023

# 1 Initial management of critically buried avalanche victims

**Time of avalanche** ____ : ____

**Provider at the head of the patient. Time of face exposure** ____ : ____

**Assess airway patency**

- ☐ Airway obstructed
- ☐ Airway patent or unknown

**Duration of burial** _____ min

☐ **≤ 60 minutes**

Presumed asphyxia

**Check for signs of life for no more than 10 seconds**[1]

**Signs of life present?**[1]

- ☐ **YES** → Avalanche victim with signs of life - see below
- ☐ **NO** → **Give five rescue breaths**

☐ **> 60 minutes**

Possible hypothermia

**Check for signs of life for up to 1 minute**[1]

**Signs of life present?**[1]

- ☐ **YES** → Avalanche victim with signs of life - see below
- ☐ **NO** → **ECG monitoring as soon as possible if available**
  - ☐ Asystole
  - ☐ VF ☐ PEA ☐ Unknown

**Avalanche victim with signs of life - see below**

**Start CPR as soon as possible**[2]

Do not start CPR if: burial duration > 60 min & obstructed airway & asystole

**ALS**

**Measure oesophageal temperature as soon as possible** __ , _ °C

**Use algorithm 2 for decision making**

**Critically buried avalanche victim with signs of life**

- ECG monitoring as soon as possible (ideally before handling or moving the patient)
- Gentle handling and consider potential trauma
- Transfer to the most appropriate hospital
- Consider transfer to an ECLS hospital for hypothermic patients with: Core temperature <30°C or ventricular arrhythmia or systolic blood pressure <90 mmHg
- The management of medical conditions which are not specific to avalanche victims (e.g. hypothermia, trauma) should follow the most recent recommendations

# 2 Decision making algorithm for advanced management of critically buried avalanche victims in cardiac arrest

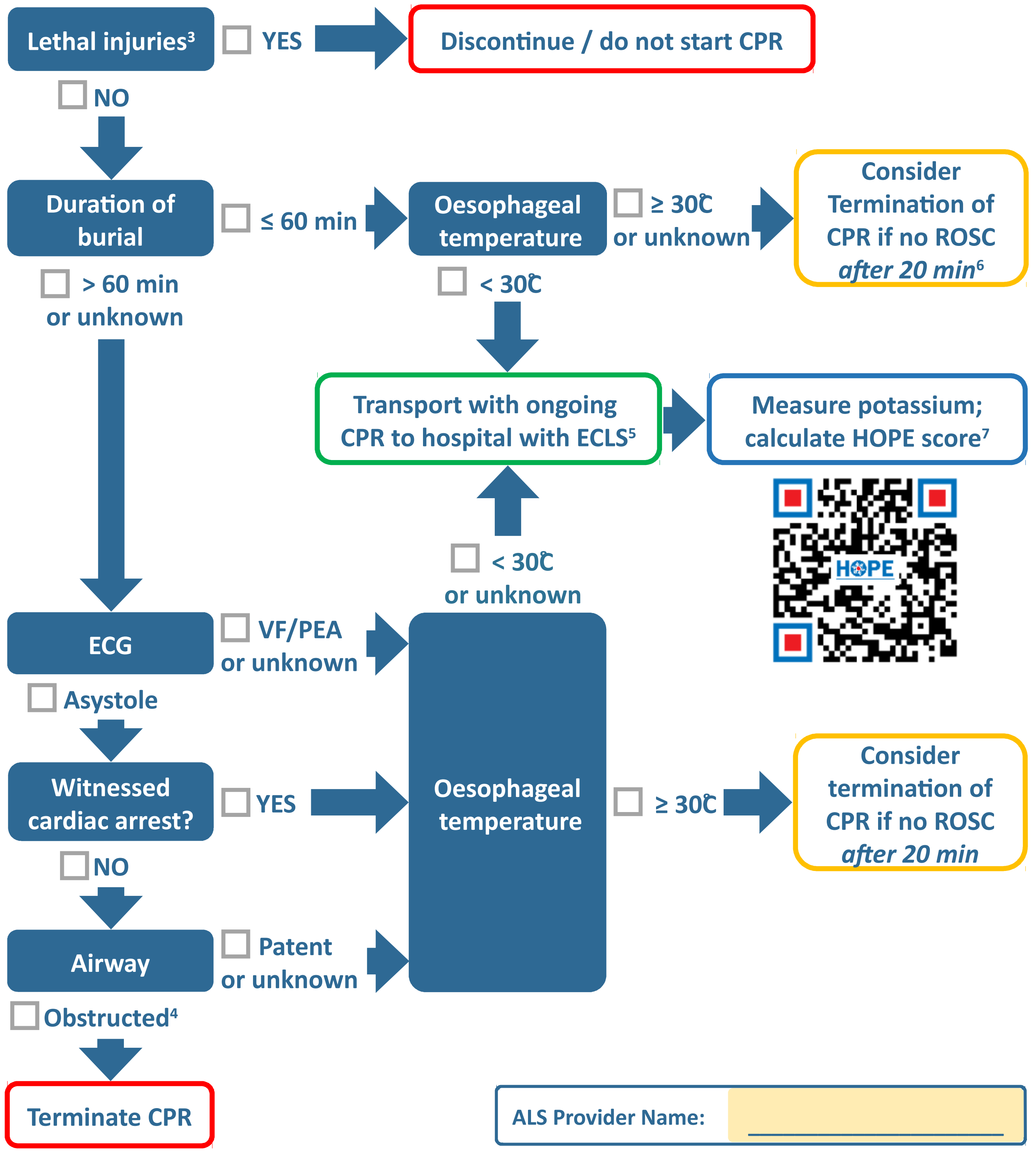


1. Signs of life include any of the following: A, V or P from AVPU (alert, responsive to verbal stimuli, responsive to pain, unresponsive) or Glasgow Coma Scale >3, any visible movement, respirations, or a palpable carotid or femoral pulse (for experienced ALS providers).
2. Standard compression / ventilation rates. Drug dose and defibrillation depending on core temperature or, if not available, burial duration. If ventricular fibrillation persists after three shocks, delay further attempts until the core temperature is ≥30°C. Withhold adrenaline if the core temperature is <30°C.
3. Assess for lethal injuries: decapitation; truncal transection; whole body decomposed. If present, do not start CPR.
4. An “obstructed” or ”blocked” airway requires both the nose and mouth to be completely filled with compact snow or debris.
5. With a deeply hypothermic patient (<28°C), if rescue is too dangerous consider delayed CPR and if transport is difficult consider intermittent CPR.
6. If core temperature measurement is not available, hypothermic CA may be considered, at the rescuer's discretion, despite a burial duration of ≤60 minutes in a victim with a patent airway and no signs of life where there is the possibility of very rapid cooling (e.g. burial during ascent, thin or small person, minimally dressed, sweating before burial).
7. In-hospital prognostication of successful rewarming in an avalanche victim should include the estimation of the survival probability using the HOPE score. If any doubt exists whether the avalanche victim may have been asphyxiated despite critical burial, the HOPE score should be calculated using the NON-ASPHYXIA option. This will reduce the risk of undertreatment. If the HOPE score cannot be determined, the combination of a potassium <7 mmol/L and a temperature <30°C may be used instead to help indicate ECLS rewarming.
